# Electrophysiological Correlates of the Effect of Task Difficulty on Inhibition of Return

**DOI:** 10.3389/fpsyg.2018.02403

**Published:** 2018-12-04

**Authors:** Ai-Su Li, Cheng-Guo Miao, Yu Han, Xun He, Yang Zhang

**Affiliations:** ^1^Department of Psychology, Soochow University, Suzhou, China; ^2^Department of Psychology, Bournemouth University, Poole, United Kingdom

**Keywords:** task difficulty, inhibition of return, event-related potential (ERP), N1, P2

## Abstract

Inhibition of return (IOR) refers to slower responses to targets that occur at a previously attended location than to those at control locations. Previous studies on the impact of task difficulty on IOR have shown conflicting results. However, these studies failed to match low-level characteristics of stimuli (e.g., size, color, and luminance) across difficulty levels, and so might have confounded the effect of task difficulty with that of stimulus characteristics. Hence, whether and how task difficulty modulates IOR remain largely unknown. This study utilized the event-related potentials (ERPs) technique in combination with a cue-target paradigm to tackle this question. Task difficulty was manipulated by changing the position of a gap in a rectangle stimulus, while stimulus size, color, and luminance were precisely matched. IOR was observed in reaction times across all difficulty levels but was found in accuracy at the medium level only. The modulation effect of task difficulty on IOR was also evident in the N1 and P2 ERP components, which showed significantly weaker IOR effects at the medium difficulty level than at the easy and hard levels. It is suggested that the modulation of IOR by task difficulty involves both perceptual and post-perceptual processes.

## Introduction

Efficiently searching for a target in a complex environment is a fundamental skill for human beings (Najemnik and Geisler, 2005; MacInnes et al., 2014). One way to maintain the search efficiency is to reduce the probability of inspecting the same location repetitively (Klein, 2000). Extensive research has demonstrated that an effect, namely inhibition of return (IOR), plays a vital role in facilitating the visual search by discouraging attention from returning to previously searched locations (Wang and Klein, 2010; Dukewich and Klein, 2015). IOR was first reported by Posner and Cohen (1984), who found that when target stimuli appeared more than 250 ms after the onset of an uninformative peripheral cue (i.e., when stimulus onset asynchrony, SOA, was larger than 250 ms), participants responded more slowly to targets at cued locations than to those at uncued locations. Numerous studies have ever since attempted to reveal IOR’s behavioral components (Zhang and Zhang, 2011; Hilchey et al., 2012), plasticity (Xu et al., 2016), and the effect of task type on IOR (Lupiáñez et al., 1997; Satel et al., 2014).

In addition to IOR, task difficulty is another crucial factor in visual search. For example, Tales et al. (2004) and Huang and Pashler (2005) found that even the same participant displayed different levels of search efficiency at different levels of task difficulty. Gaspelin et al. (2016) and Barras and Kerzel (2017) revealed the critical role of search difficulty in the expression of attentional capture by irrelevant stimuli (e.g., onset cues or color distractors). Moreover, recently Pomplun et al. (2013) found that participants’ eye movements and processing strategies changed with task difficulty. Starting with information available around the fixation point in easy tasks, the participants used a from-left-to-right and from-top-to-bottom strategy to process information in difficult tasks. Despite widespread understanding regarding the influence of task difficulty in visual search, only a limited amount of literature has investigated the impact of task difficulty on IOR, which plays an essential role in maintaining visual search efficiency (Klein, 2000). Several studies have suggested that task difficulty might influence IOR. For example, Lupiáñez et al. (2001) observed that IOR was much smaller in a difficult task (i.e., discriminating between M and N; the IOR effects were 0 and 8 ms with SOAs of 700 and 1,000 ms, respectively) than in an easy task (i.e., discriminating between X and O; the IOR effects were around 36 and 30 ms ^1^ with SOAs of 700 and 1,000 ms, respectively). Similarly, Castel et al. (2005) observed significant smaller IOR for a perceptually degraded target (an average IOR effect of 6 ms across 11 SOAs) than for an easy-to-detect (large and bright) target (an average IOR effect of 18 ms across 11 SOAs). However, Cheal and Chastain (2002) used similar manipulations but did not find any influence of task difficulty on IOR. The effect of task difficulty on IOR, therefore, has to date remained unclear.

A careful examination of these studies revealed substantial differences in the target stimuli across tasks of varying difficulty levels, including shape, size, brightness, and color. Therefore, it is hard to determine whether a study’s findings were due to task difficulty manipulation or imbalance in these low-level properties. It is also worth noting that, although task difficulty was often corroborated by longer reaction times (RTs) in hard tasks than in easy tasks, accuracy data rarely validated task difficulty manipulations (cf. Lupiáñez et al., 2001). For instance, in the studies by Cheal and Chastain (2002) and Castel et al. (2005), the accuracy only showed differences of about 2% between task difficulty levels. Therefore, the diverse result patterns in these previous studies were likely to be associated with the varied task-difficulty manipulations, some of which might have been imperfect.

In addition to behavioral measures, increasingly more studies have used event-related potential (ERP) to investigate the neural correlates of IOR (McDonald et al., 1999; Prime and Ward, 2004, 2006; Wascher and Tipper, 2004; Chica and Lupiáñez, 2009; Prime and Jolicoeur, 2009; Zhang et al., 2012; Martín-Arévalo et al., 2014). In contrast to behavioral measures, ERP has a high temporal resolution and can more precisely track the time course of cognitive processes (Luck, 2014). Different ERP components are typically closely associated with various information-processing stages (Luck and Kappenman, 2012). For instance, an attention effect in the P1 component is a good indicator of an early feed-forward sensory information processing (Zhang and Luck, 2009). By contrast, an attention effect in the N1 component primarily reflects a visual discrimination process (Vogel and Luck, 2000). Indeed, many ERP studies on IOR revealed IOR modulations in P1 and N1 (Prime and Ward, 2006; Prime and Jolicoeur, 2009; Zhang et al., 2012; Satel et al., 2013, 2014; see Martín-Arévalo et al., 2016, for a comprehensive review). A cueing effect in P2 (also known as Nd or Nd250) was also observed. Although this effect is generally believed to represent post-perceptual processing, controversy remains regarding its specific implications (Martín-Arévalo et al., 2016). Some researchers believe that this P2 effect is an electrophysiological index of IOR *per se* (Satel et al., 2014; Xu et al., 2016), whereas others suggested that it may only reflect complementary facilitation of the perceptual inhibition at previous cued locations (Wascher and Tipper, 2004).

**FIGURE 1:**
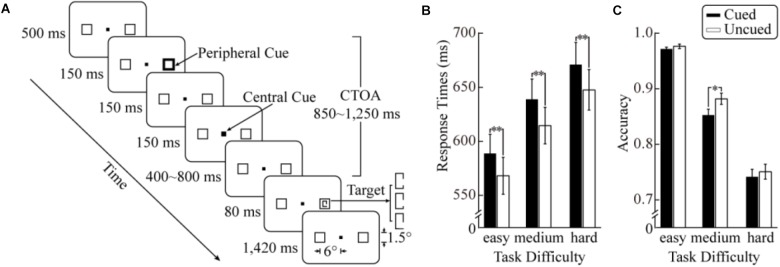
Trial sequence and behavioral results of the current study. **(A)** Schematic diagram of the stimuli and procedure. The background was gray and the central and peripheral cues white. **(B)** Reaction times (RTs) to target stimuli as a function of cueing and task difficulty. **(C)** Results in accuracy. The error bars denote ±1 SEM; ^∗^*p* < 0.01, ^∗∗^*p* < 0.001.

In the current study, we adopted the ERP technique and the cue-target paradigm to investigate the influence of task difficulty on IOR. We manipulated the target discrimination difficulty using a psychophysical procedure while matching low-level physical features of targets (size, color, and brightness) across difficulty levels. In a trial, following a peripheral cue, a target stimulus randomly appeared at the cued or uncued location. The target stimulus was a box with a small gap on the side furthest away from fixation. Participants were asked to discriminate whether the gap was above or below the horizontal meridian (Figure 1A). In this paradigm, the distance between the gap and the horizontal meridian will determine the discrimination difficulty. In the current study, this difficulty was precisely maintained at three levels respectively corresponding to accuracy of 70%, 85%, and 100%, separately measured with a psychophysical procedure for each participant. If task difficulty has an effect on IOR, we predicted that the IOR effect (i.e., the RT difference between cued and uncued conditions) would differ across the three task difficulty levels. The ERP data would help to reveal the neural correlates of the IOR modulation. If task difficulty influences IOR at the sensory-processing stages, the cueing effect in P1 and/or N1 (indicators of sensory processing) would be modulated by task difficulty. If the IOR modulation by task difficulty takes place after sensory processing, the corresponding effect would be seen in the P2 component (an indicator of post-sensory processing).

## Materials and Methods

### Participants

Eighteen paid, volunteers who were naïve to the experiment (13 females, 5 males; 20.2 ± 1.6 years old) from Soochow University participated. All participants had normal or corrected-to-normal vision and no experience in similar experiments. Three participants were excluded from analysis due to excessive electroencephalogram (EEG) artifacts. According to a simulation study by Boudewyn et al. (2018), the 15 usable participants together with 312 trials per condition provided a statistical power much higher than 0.8 for within-participants designs, with a difference of 0.5 μV (see Figure 8 in their study). All participants gave written informed consent following the standard of the Declaration of Helsinki. The study was approved by the Ethics Committee of Department of Psychology, Soochow University.

### Experimental Design

The current study used a 3 (task difficulty) × 2 (cueing) within-participant design. There were three levels of task difficulty (hard, medium, and easy) and two levels of cueing (cued and uncued). In cued trials, the peripheral cue and the target appeared at the same location; in uncued trials, the peripheral cue and the target appeared at different locations.

### Apparatus, Stimulus, and Procedure

The experiment program was generated in E-Prime 2.0 (Psychology Software Tools, Inc., Sharpsburg, PA, United States), and run on a Lenovo ThinkCentre (M4300T) computer equipped with an nVIDIA GeForce GT 630 graphics card. The stimuli were displayed against a gray background (11.95 cd/m^2^) on a 22-inch cathode-ray tube monitor (Philips 202P40, 100-Hz refresh rate, resolution of 1024 × 768 pixels). Participants were seated in a dimly lit and sound-attenuated room at a viewing distance of 80 cm. The participants responded to the stimuli with a seven-key gamepad (Microsoft SideWinder X04-97602).

An example of the stimuli and trial procedure used in the main experiment is shown in Figure 1A. (1) Each trial began with the display of a black fixation square at the center of the screen (0.2° × 0.2°), flanked by two black boxes (1.5° × 1.5°) 6° away (center to center) to the left and right of the fixation. (2) After 500 ms, one of the peripheral boxes was brightened and thickened with equal probability for 150 ms as a peripheral cue, which did not provide any probabilistic information about the location of a forthcoming target. (3) 150 ms after the offset of the peripheral cue, the central fixation square was whitened and thickened for 150 ms as a central cue, forcing the attention back to the fixation location. According to past studies (Prime and Jolicoeur, 2009; Martín-Arévalo et al., 2014; Xu et al., 2016), the central cue is important for obtaining robust IOR effects in discrimination tasks. (4) After a random delay of 400 to 800 ms – the randomization was employed to eliminate the overlap of cue-evoked and target-evoked ERP activities (Woldorff, 1993) – the target was presented for 80 ms in one of the peripheral boxes with equal probabilities (and also at the cued or uncued location with equal probabilities). The target was a rectangle in a portrait layout (0.5° × 1°) with a gap (0.4°) on the side furthest away from the fixation. The distance between the gap and the horizontal meridian could be randomly one out of three possible values (corresponding to the three task difficulty levels). The values of these distances were individually determined for each participant with a psychophysical procedure immediately before the main experiment. (5) After another delay of 1,420 ms, the trial ended with the disappearance of the target. Participants were required to discriminate the position of the gap (above or below the horizontal meridian) and report by pressing one of two keys (“left” and “right” keys) as quickly and accurately as possible. The mapping between gap positions and responding keys was counterbalanced across participants. Before the experiment, participants were explicitly informed that the peripheral cues did not predict the location of the target stimuli.

To improve the signal-to-noise ratio of the ERP data (i.e., getting more usable trials in each experimental condition) and to prevent fatigue, each participant completed the main experiment in two 2.5-h sessions (including preparation time) over two consecutive days. Each session had thirteen 72-trial blocks. Overall, each participant performed 1,872 trials (312 trials for each condition). Each session was preceded by approximately 20 practice trials where feedback was provided on participants’ responses.

Before the first session of the main experiment, the participants spent approximately 30 min to complete the psychophysical procedure. The procedure was similar to that of the main experiment except that there was no peripheral or central cue, and that there were ten possible distances (either above or below the horizontal meridian) between the gap and the horizontal meridian (corresponding to 10 difficulty levels). The participants were asked to discriminate and report whether the gap was located above or below the horizontal meridian. This psychophysical procedure consisted of six 80-trial blocks, leading to 48 trials for each difficulty level (including positions above and below the horizontal meridian). With the data from the psychophysical procedure, for each participant, the gap distances used at the three difficulty levels (corresponding to 70%, 85%, and 100% accuracy) in the main experiment were determined by fitting a cumulative normal distribution function to each participant’s performance using the maximum likelihood curve fitting method (Wichmann and Hill, 2001). Across participants, the gap-meridian distances used in the main experiment for the hard, medium, and easy conditions were 0.083 ± 0.025°, 0.148 ± 0.048°, and 0.300°, respectively.

### EEG Data Collection and Pre-processing

EEG and electrooculogram (EOG) data were recorded continuously from 64 Ag/AgCl electrodes (Quick-Cap) positioned according to the extended international 10–20 system. Continuous EEG and EOG were amplified with SynAmps^2^ amplifiers (Compumedics Neuroscan, Charlotte, NC, United States) in the AC mode with a physical band-pass filter of 0.05–100 Hz and digitized at a rate of 1,000 Hz. All electrodes were referenced to the left mastoid. The EOG was recorded horizontally from the outer canthi of both eyes (hEOG) and vertically from above and below the left eye (vEOG). Electrode impedances were kept below 5 KΩ.

After manual removal of the apparent artifacts, a zero-phase-shift band-pass filter of 0.1–30 Hz was applied, and the ocular artifacts were corrected by a linear regression method (Gratton et al., 1983). Subsequently, data were digitally re-referenced to the mean voltage of electrical activities recorded at the two mastoid electrodes and were segmented into epochs starting 200 ms before target onset and ending 800 ms after target onset. After baseline correction, segments with eye movements or eye blinks within the range between -200 ms and 400 ms (identified using a moving-window procedure carried out on the vEOG and hEOG channels with a threshold of 35 μV, moving step of 4 ms; Lopez-Calderon and Luck, 2014; Luck, 2014), or artifacts at any of the EEG channels (amplitudes exceeding ±75 μV) were excluded from further analyses. Overall, approximately 5.4%, 6.6%, and 7.0% of the data were rejected from the ERP analysis, leaving 432 (69.2%), 500 (80.2%), and 564 (90.4%) trials for hard, medium and easy conditions, respectively (the minimal numbers of remaining trials across participants were 392, 462, and 519 for the hard, medium, and easy conditions, respectively).

## Data Analysis and Results

### Behavioral Data Trimming

For each condition of each participant, RTs less than 100 ms (less than 0.1% of total trials) or more than 2.5 standard deviations away from the mean of that condition (around 2% of total trials) were excluded from analysis. Subsequently, mean RTs of the trials with correct responses and accuracy were submitted to repeated-mesaures analyses of variance (ANOVAs) with factors of task difficulty (easy, medium, and hard) and cueing (cue vs. uncued).

#### RTs

Figure 1B shows the mean RTs as a function of cueing for each task difficulty level. The ANOVA revealed a significant main effect of task difficulty, *F*(2,28) = 99.23, *p* < 0.001, $\eta_{p}^{2}$ = 0.88. As task difficulty increased, RTs increased significantly (578 ms, 627 ms, and 659 ms for the easy, medium, and hard conditions, respectively). The main effect of cueing was also significant, *F*(1,14) = 116.19, *p* < 0.001, $\eta_{p}^{2}$ = 0.89, with slower responses in the cued condition (633 ms) than in the uncued condition (610 ms). The task difficulty × cueing interaction did not reach statistical significance (*F* < 1).

#### Accuracy

The mean accuracies in the six experimental conditions are shown in Figure 1C. The ANOVA showed a significant main effect of task difficulty, *F*(2,28) = 191.12, *p* < 0.001, $\eta_{p}^{2}$ = 0.93 (97.4%, 86.7%, and 74.6% in the easy medium and hard conditions, respectively), suggesting that the manipulation of task difficulty was effective. The main effect of cueing was also significant, *F*(1,14) = 14.56, *p* = 0.002, $\eta_{p}^{2}$ = 0.51. The accuracy was higher in the uncued condition (87.0%) than in the cued condition (85.5%). More importantly, a significant task difficulty × cueing interaction was observed, *F*(2,28) = 3.89, p = 0.032, $\eta_{p}^{2}$ = 0.22. Additional simple effect analyses demonstrated that a significant cueing effect was only observed in the medium condition, *F*(1,14) = 14.59, *p* = 0.002, with a higher accuracy in the uncued condition (88.2%) than in the cued condition (85.3%). An additional analysis directly comparing the cueing effects across difficulty levels showed that the cueing effect of accuracy in the medium condition (3.0%) was significantly larger than that in the easy (0.5%, two-tailed *t*_14_ = 2.85, *p* < 0.05) and hard conditions (0.9%, two-tailed *t*_14_ = 2.08, *p* = 0.056).

It is worth noting that ANOVA may not be an appropriate method for accuracy data analysis because accuracy data is inherently categorical (a correct/wrong response in each trial) (Dixon, 2008; Jaeger, 2008)^2^. Therefore, to provide additional converging evidence, mixed logit models (a type of generalized linear mixed models; Dixon, 2008; Jaeger, 2008) with a maximal random effects structure (Barr, 2013; Barr et al., 2013) was carried out to analyze the accuracy data. The significance of the main effects and interactions was tested by comparing the model including the effects against a model without the effects using a χ^2^ test (the *compare* function in MATLAB 2015b; The MathWorks, Inc., Natick, MA, United States). The mixed logit models suggested a significant main effect of task difficulty, χ^2^(2) = 45.55, *p* < 0.001 (97.4%, 86.7%, and 74.6% in easy, medium, and hard condition, respectively). The main effect of cueing failed to reach significance, χ^2^(1) = 2.83, *p* = 0.09. The task difficulty × cueing interaction was confirmed to be significant, χ^2^(2) = 6.27, *p* < 0.05. Follow-up simple effect analyses identified a significant cueing effect in the medium condition only, χ^2^(2) = 11.46, *p* < 0.001.

### EEG Data

According to previous studies (Wascher and Tipper, 2004; Zhang et al., 2012; Xu et al., 2016) and in line with visual inspection of the topographic maps of the mean cueing effect across all task difficulty conditions (bias in determining analysis parameters was avoided; see Kriegeskorte et al., 2009), the P1 (PO7, PO5, PO6, and PO8; 108–128 ms), N1 (CP1, CPz, CP2, P1, Pz, and P2; 140–180 ms), P2 (C1, Cz, C2, CP1, CPz, CP2, P1, Pz, and P2; 300–330 ms), and P3 (C1, Cz, C2, CP1, CPz, and CP2; 400–600 ms) components were quantified at the selected electrode and analysis windows. For each ERP component, the analysis window was centered around the time point showing maximal difference between the cued and uncued conditions. Mean ERP amplitudes were analyzed with repeated-measures ANOVAs, with factors of task difficulty (easy, medium, and hard) and cueing (cued and uncued). Greenhouse-Geisser correction procedure of *p*-values was applied (reported as *p_c_*) when the sphericity assumption was violated.

Additionally, to prevent potential selection bias in the analysis windows and to provide converging evidence, we employed growth curve analysis (GCA) with maximal random effects structure (Barr, 2013; Mirman, 2014) for full-window analyses (i.e., throughout the durations of the entire components being analyzed). GCA is a multi-level regression technique developed for analysis of time courses or longitudinal data (Mirman, 2014; West et al., 2014). While in traditional analysis EEG data are treated as discrete variables (mean amplitudes of some specific time-window waveforms), GCA treats time-course data as a continuous variable and thus has a higher statistical power. Following the suggestions by Barr (2013) and Mirman (2014), the nested-model comparison was used to examine the significance of task difficulty. Namely, the two models with and without the factor of task difficulty were compared to determine the contribution of task difficulty. These GCA analyses were carried out on the difference waveforms between the cued and uncued conditions in the time ranges of the P1 (100–150 ms), N1 (140–200 ms) and P2 (230–390 ms) components. The *fitlme* and *compare* functions in MATLAB were used for model estimation and comparison respectively.

#### P1

**FIGURE 2:**
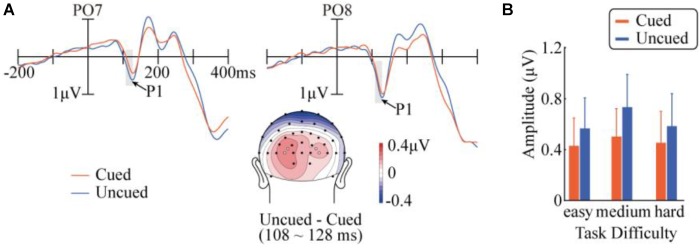
Results in the P1 component. **(A)** Target-evoked ERP waveforms of cued and uncued conditions at PO7 and PO8, and the topographic map of the difference between cued and uncued conditions. The topographic map was generated with a customized MATLAB function based on EEGLAB’s *topoplot* function (Delorme and Makeig, 2004), and is available at https://github.com/yangzhangpsy/3dtopoplot. Analyzed electrode locations are marked as white dots in the map. **(B)** Mean amplitudes across task difficulty and cueing conditions over the four analyzed electrode locations.

Figure 2 demonstrates the ERP waveforms, topographic maps, and mean amplitudes in the P1 component. A repeated-measures ANOVA of 3 (task difficulty) × 2 (cueing) × 4 (electrode) revealed a main effect of cueing, *F*(1,14) = 5.06, *p* = 0.041, $\eta_{p}^{2}$ = 0.27, with a smaller P1 amplitude in the cued condition (0.46 μV) than in the uncued condition (0.63 μV). The main effect of the electrode was also significant, *F*(3,42) = 4.84, *p*_c_ = 0.044, $\eta_{p}^{2}$ = 0.26). The interaction between cueing and task difficulty was not significant, *F* < 1. None of the other main effects or interactions approached significance.

#### N1

**FIGURE 3:**
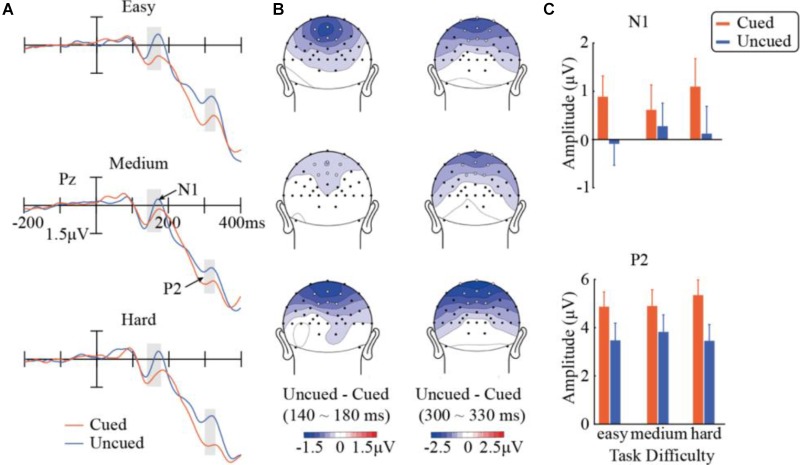
Results in the N1 and P2 components. **(A)** ERP waveforms evoked by the target stimuli across the three task-difficulty levels and the cued and uncued conditions at Pz showing N1 and P2. **(B)** Topographic maps of differences between cueing conditions within the analysis windows of N1 (140–180 ms) and P2 (300–330 ms). White dots represent the electrode locations used in the analysis. **(C)** Mean amplitudes of N1 (over six analyzed electrode locations) and P2 (across nine electrode locations).

Results in the N1 component are illustrated in Figure 3. A repeated-measures ANOVA of 3 (task difficulty) × 2 (cueing) × 6 (electrode) found a significant main effect of cueing, *F*(1,14) = 19.83, *p* < 0.001, $\eta_{p}^{2}$ = 0.59, with significantly more negative amplitudes in the uncued condition (0.10 μV) than in the cued condition (0.87 μV). A significant task difficulty × cueing interaction was obtained, *F*(2,28) = 5.02, *p* = 0.014, $\eta_{p}^{2}$ = 0.26. Further simple effect analyses indicated that the cueing effect was only significant when the task was easy [*F*(1,14) = 31.42, *p* < 0.001] or hard [*F*(1,14) = 26.64, *p* < 0.001], but did not reach significance at the medium difficulty level [*F*(1,14) = 1.51, *p* > 0.23]. A direct comparison between the cueing effects across difficulty levels confirmed that the magnitude of the cueing effect in N1 was significantly smaller in the medium condition (-0.339 μV) than those in the easy (-0.976 μV, two-tailed *t*_14_ = 2.29, *p* < 0.05) and hard conditions (-0.975 μV, two-tailed *t*_14_ = 2.67, *p* < 0.05). Other main effects or interactions did not reach significance.

#### P2

Figure 3 summarizes the results in P2 as well. A repeated-measures ANOVA of 3 (task difficulty) × 2 (cueing) × 9 (electrode) observed a main effect of cueing, *F*(1,14) = 28.10, *p* < 0.001, $\eta_{p}^{2}$ = 0.67). Compared with the uncued condition (3.59 μV), the cued condition evoked a stronger P2 component (5.04 μV), showing a significant Nd effect (P2_Uncued_ – P2_Cued_). More importantly, the task difficulty × cueing interaction was also significant, *F*(2,28) = 4.79, *p* = 0.016, $\eta_{p}^{2}$ = 0.26. Follow-up simple effect analyses showed that the cueing effect was significant at all three task-difficulty levels. The cueing effect marginally differed across the difficulty levels, *F*(1,14) = 4.58, *p_c_* = 0.0504, $\eta_{p}^{2}$ = 0.25, with the medium condition (1.07 μV) having a weaker cueing effect than the easy (1.40 μV) and hard conditions (1.90 μV). The interactions of task difficulty × electrode [*F*(16,224) = 8.75, *p_c_* < 0.001, $\eta_{p}^{2}$ = 0.39] and cueing × electrode [*F*(8,112) = 14.3, *p_c_* < 0.001, $\eta_{p}^{2}$ = 0.51] were also significant. None of the other main effects or interactions were significant.

#### P3

**FIGURE 4:**
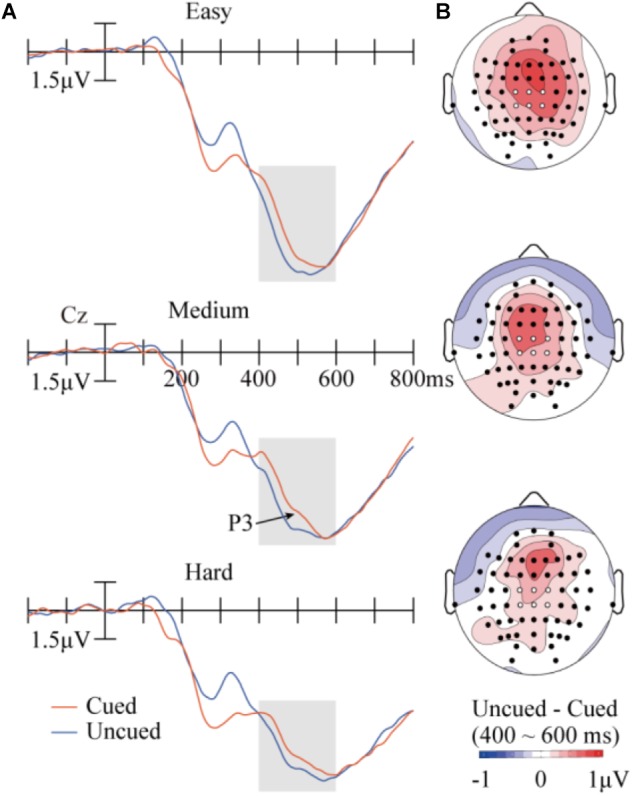
Results in the P3 component. **(A)** Target-evoked ERP waveforms (at Cz) at the three difficulty levels across the cued or uncued conditions. The six analyzed electrode locations are shown as white dots in the maps. **(B)** Topographic maps of the differences between cueing conditions.

Figure 4 illustrates the ERP results in P3. The data was analyzed with a repeated-measures ANOVA of 3 (task difficulty) × 2 (cueing) × 6 (electrode). A main effect of task difficulty was found, *F*(2,28) = 35.91, *p* < 0.001, $\eta_{p}^{2}$ = 0.72, with P3 showing weaker amplitudes when difficulty increased (9.94 μV, 8.05 μV, and 7.36 μV for the easy, medium, and hard conditions, respectively). The main effect of cueing was also significant, *F*(1,14) = 19.80, *p* < 0.001, $\eta_{p}^{2}$ = 0.59, with larger P3 amplitudes in the uncued condition (8.72 μV) than in the cued condition (8.18 μV). The interaction between task difficulty and cueing was far from significant, *F*(2,28) = 0.63, *p* = 0.54, $\eta_{p}^{2}$ = 0.043. None of the other main effects or interactions approached significance.

#### GCA Results for EEG

GCA modeling was used to confirm the results of the traditional analyses reported above, specifically for the P1, N1, and P2 components in which cueing effects were observed. The GCA results were consistent with the results in the ANOVAs: task difficulty significantly influenced the cueing effect in N1 and P2 [χ^2^(6) = 14.65, *p* < 0.05 and χ^2^(6) = 13.62, *p* < 0.05, respectively] but did not modulate the cueing effect in P1 [χ^2^(6) = 9.74, *p* = 0.14].

## Discussion

Although several previous studies have investigated the impact of task difficulty on IOR, those studies mostly used target stimuli that were substantially different from one another in some features, such as size, color, and brightness (Cheal and Chastain, 2002; Castel et al., 2005). Therefore, it is hard to determine whether the modulation effects observed in IOR resulted from the applied task difficulty modulations or the confounding factors of visual features (Lupiáñez et al., 2001; Cheal and Chastain, 2002; Castel et al., 2005). The current study investigated the effect of task difficulty on IOR by precisely matching the low-level stimulus features including size, color, and brightness across task difficulty levels. The only remaining difference was the distance between the gap on the target stimulus and the horizontal meridian, which was unlikely to impact on the strength of perceptual processing in the brain and the associated behavioral responses. To more reliably manipulate the task difficulty level, a separate psychophysical procedure was employed and, therefore, the stimuli were meticulously selected to reach the targeted accuracy. Although our RT data did not show any influence of task difficulty on IOR, the accuracy data clearly exhibited a modulation of IOR by task difficulty. Our findings imply that the reason Cheal and Chastain (2002) failed to observe an influence of task difficulty on IOR might be the relatively weaker task-difficulty manipulation compared to those used by the current study and Lupiáñez et al. (2001). It is suggested that task-difficulty manipulation needs to be carefully designed in future studies.

It is interesting that the current study showed the interaction between task difficulty and cueing in accuracy, but not in RTs. One possibility could be that the participants in the current study placed much emphasis on accuracy, leading to a higher sensitivity to task difficulty in accuracy data than in RTs. Unfortunately, the current design did not enable us to explore the characteristics of these effects in detail, because the current study only examined a single point in the speed-accuracy function (Wickelgren, 1977). In the future, these characteristics could be studied by introducing a speed-accuracy tradeoff procedure to the current paradigm.

### Modulation Effect of Task Difficulty on IOR in Accuracy

The current study found that task difficulty had a modulating effect on IOR in accuracy. Interestingly, the cueing effect did not change linearly across the task difficulty levels. Specifically, the IOR effects across difficulty levels exhibited an inverted-U pattern, only reaching significance at the medium difficulty level. This finding possibly reflects a combined effect of top-down and bottom-up factors. In the hard condition, task performance was mainly constrained by the limited inputs (bottom-up factors). Therefore, allocating additional resources would not significantly improve task performance because it was the low differentiability of the stimuli rather than the strength of stimuli *per se* that caused the difficulty. This argument is also supported by Kanai et al. (2006), who found that the physiologically reduced stimuli (rendered invisible by a continuous flash suppression method) entirely abolished the spatial attention effect.

In the easy condition of the current study, the participants could quickly complete the task without investing additional resources, thus leaving little space for IOR to arise. Consistent with this speculation, Chen et al. (2008) manipulated task difficulty by changing the stimulus similarity (comparable to the manipulation used in the current study), and revealed a stronger spatial attention effect in the hard condition (accuracy of 86%, which is comparable to the performance in our medium difficulty condition) compared to the easy condition accuracy of 98%, which is comparable to the performance in our easy condition.

Therefore, it is likely that the cognitive system strategically assigned relatively more resources to the medium task difficulty level because resources invested in this condition would pay the best interest. In line with this assumption, some studies have revealed top-down factors that could affect the IOR effect, such as expectancy (Jefferies et al., 2005; Spalek, 2007) and appearance frequency (Lupiáñez et al., 2007). For example, Jefferies et al. (2005) concluded that expectancy plays a crucial role in exhibiting IOR with occluded objects. In another example, Lupiáñez et al. (2007) found that the IOR effect was affected by the frequency of target stimuli. However, it should be noted that, without direct evidence, this explanation is speculative and needs to be tested in the future.

### Temporal Characteristics of the Task-Difficulty Modulation on IOR

The ERP results in the current study have significant implications for understanding the time course of task difficulty’s influence on IOR. In contrast to behavioral measures, which only reflect the sum of all effects across all information-processing stages and are unable to provide the temporal profile over multiple information-processing stages, the ERP technique has a high temporal resolution and is ideal for evaluating the temporal dynamics of cognitive processes (i.e., determining the processing stage during which a particular effect occurs; Luck, 2014). Consistent with previous ERP studies on IOR (Prime and Ward, 2004, 2006; Prime and Jolicoeur, 2009; Zhang et al., 2012; Satel et al., 2014), the current study has observed significant cueing effects in P1 (which reflects early sensory processing), N1 (sensitive to visual discrimination processing), and P2 (which reflects post perceptual processing). More importantly, different influence patterns of task difficulty in these components were discovered. Task difficulty did not modulate the cueing effect in P1, an indicator of the early sensory analysis of visual stimuli (Luck and Hillyard, 1994, 1995; Zhang and Luck, 2009; Luck and Kappenman, 2012). This modulation effect, however, was significant in the cueing effect slightly later in the N1 time range, a stage reflecting the discrimination processing of visual stimuli (Luck, 2014; Vogel and Luck, 2000). A similar modulation effect was detected in the P2 component (Nd250), which is considered a most likely indication of attention factors in IOR (Wascher and Tipper, 2004; Xu et al., 2016). In sum, our current results suggest that the influence of task difficulty on IOR occurs at multiple visual processing stages, but not during the early stage of sensory processing. Our findings agree with Fedota et al. (2012), who also found that the modulation of task difficulty on stimulus processing occurs in processing stages later than early P1.

### Effects of Task Difficulty and Cueing in P3

It has been proposed that the amplitude of P3 is related to the amount of information transmitted that depends on equivocation (the posterior uncertainty about having correctly identified a target) and attention (Johnson, 1988; Kok, 1997), and is thus inversely proportional to the task difficulty (Kok, 1997; Polich, 2012). In the present study, the effect of task difficulty on the P3 amplitude is in line with this interpretation. The rise in task difficulty from easy to hard increased uncertainty about having correctly processed targets, thereby decreasing the amplitude of P3 elicited by the target stimuli (Kok, 1997). This result is also consistent with many previous ERP studies in that, compared with low difficulty conditions, targets in high difficulty conditions evoked smaller P3 amplitudes (Kok, 1997, 2001; Fedota et al., 2012; Polich, 2012).

Also compatible with the information transmission hypothesis (Johnson, 1988; Kok, 1997) about the P3 amplitude, the current results showed lower P3 amplitudes in the cued than the uncued condition. Previous ERP studies have found weaker neural representations of visual stimuli in the cued condition than in the uncued condition (McDonald et al., 1999; Klein and Dick, 2002; Prime and Ward, 2004, 2006; Zhang et al., 2005; Prime and Jolicoeur, 2009; Zhang et al., 2012; Xu et al., 2016). According to the information transmission theory, a weaker representation would lead to less information transmission, thereby eliciting a smaller P3 amplitude.

The significant cueing effect in the P3 amplitude observed in the current study is also compatible with traditional attention theories and a more recent segregation-integration theory of IOR (Lupiáñez and Milliken, 1999; Lupiáñez et al., 2007, 2013; Funes et al., 2008). The theories of visual attention suggest that IOR originates in a lack of attention at the cued location (Klein and Dick, 2002; McDonald et al., 2009; Pierce et al., 2017). Thus, the target stimuli at the cued location are expected to have a weaker representation and thus evoked a smaller P3 (Kok, 1997). The segregation-integration theory of IOR has been developed by Lupiáñez and Milliken (1999), Lupiáñez et al. (2007), and Funes et al. (2008) on the basis of the object file framework (Kahneman et al., 1992). According to this theory, IOR depends on two independent processes of integration and separation. In the cued condition, the cognitive system tends to integrate the target stimulus into the object file (i.e., representation) triggered by the cue, whereas, the cognitive system tends to create a new object file (representation) in the uncued condition. Given that integrating information only requires rewriting part of the information and that a new representation requires more information transmission, this theory also predicts that the P3 component should be stronger in the uncued condition.

Interestingly, although the current study found significant main effects of task difficulty and cueing in P3, the interaction of these two factors was not significant. One possibility might be that the P3 amplitude reflects multiple processing processes, and might involve multiple brain areas (e.g., the prefrontal, parietal, and/or temporal lobes). If we assume that both IOR and task difficulty could have effects on different stages of information processing, which all contribute to the neural activities reflected in P3, a lack of interaction is not unreasonable. However, it should be noted here that this explanation is speculative and needs to be explored in the future.

The observation of smaller P3 amplitudes in the cued condition successfully replicated findings in several previous ERP studies on IOR. For instance, in the study by Pan et al. (2017), the amplitude of P3 was considerably reduced in the cued condition. However, there are some conflicting observations in the literature. In the studies of McDonald et al. (1999) and Chica and Lupiáñez (2009), it appeared that the P3 amplitude was greater in the cued condition than in the uncued condition. These discrepancies in results could be due to the difference of task setting among these studies. Pan et al. (2017) and the current study both used a discrimination task, while McDonald et al. (1999) and Chica and Lupiáñez (2009) used detection tasks. Consistent with this interpretation, although Chica and Lupiáñez (2009) observed a strengthened P3 in the cued condition in their detection task (see Figure 3A in their study), the results from their discrimination task resembled exactly our current data (i.e., stronger P3 in the uncued than the cued condition). Another explanation of these conflicting results could be the difference in difficulty levels determined by the task setting. Although the current study administrated three levels of task difficulty, the stimuli in these three conditions were randomly presented in the experiment. As a result, the overall task difficulty was further increased because the participants were unable to predict the difficulty level of the forthcoming stimuli. Thus, the participants applied the same level of attention engagement and strategies to manage all three difficulty levels. Consequently, our task was more challenging than those used in previous studies (McDonald et al., 1999; Chica and Lupiáñez, 2009), leading to different findings regarding the cueing effect in P3. These explanations, however, are speculative and require verification by future studies.

## Conclusion

The IOR effect is subject to the influence of task difficulty. However, this influence is only reflected in accuracy, not in the RTs. The modulation effect of task difficulty on IOR takes place at the perceptual and post-perceptual processing stages after the early stage of sensory processing.

## Data Availability

The dataset of this study is available at https://github.com/yangzhangpsy/taskDifficultyAndIOR.

## Author Contributions

YZ conceptualized and designed the study. A-SL, C-GM, and YH acquired the data. A-SL and C-GM analyzed the data. A-SL and YZ interpreted the data and drafted the manuscript. XH polished the manuscript.

## Conflict of Interest Statement

The authors declare that the research was conducted in the absence of any commercial or financial relationships that could be construed as a potential conflict of interest.

## Footnotes

1 Lupiáñez et al. (2001) did not report the IOR effects. The values reported here were extracted from Figure 5 in Lupiáñez et al. (2001) with WebPlotDigitizer (https://automeris.io/WebPlotDigitizer).

2 We would like to thank Dr. Raymond Klein for pointing this issue out.
